# Humanity Restoration Project: Bridging the Age Gap through Hands-On Intergenerational Learning

**DOI:** 10.1093/geroni/igaf122.3297

**Published:** 2025-12-31

**Authors:** Renee Beard, Lindsay Riordan, Katherine Meszaros, Emma Schultz

**Affiliations:** College of the Holy Cross, Worcester, Massachusetts, United States; College of the Holy Cross, Worcester, Massachusetts, United States; College of the Holy Cross, Worcester, Massachusetts, United States; St. Mary Health Care Center, Worcester, Massachusetts, United States

## Abstract

Intergenerational learning is thought to be an effective strategy for disrupting ageist stereotypes youth may hold and addressing the social isolation of participants. For the past 5 years, “The Humanity Restoration Project” has studied the benefits of intergenerational learning between college-aged students and nursing home residents and staff using Anne Basting’s improvisational storytelling method TimeSlips™ (TS) and the related “beautiful questions”. The central goals of the study include exposing students to intergenerational conversations, countering ageist and ableist social assumptions, and supporting community members living and working in institutional care settings. Both residents’ quality of life and staff work satisfaction have been demonstrated to improve during arts-based interventions and intergenerational programming. We collected data through observations, written student reflections, student oral presentations, pre- and post-test surveys and semi-structured individual interviews and focus groups. Students report improved ability to imagine themselves as future old people, a reduction of negativity/stigma about aging, and genuine respect for and identification with older adult participants. Residents were more engaged and felt valued through helping “teach” young people about aging. Like students, the project allowed staff to see residents in a new, more positive and personal light. Intergenerational learning has the dual benefit of disrupting negative views of aging and older people while simultaneously providing meaningful connection and social interactions. It also has the emancipatory potential - for students and staff alike - of restoring the humanity to some of the most disenfranchised among us: older adults living in nursing homes.

